# Boron-lead multiple bonds in the PbB_2_O^–^ and PbB_3_O_2_^–^ clusters

**DOI:** 10.1038/s42004-022-00643-1

**Published:** 2022-03-03

**Authors:** Wei-Jia Chen, Teng-Teng Chen, Qiang Chen, Hai-Gang Lu, Xiao-Yun Zhao, Yuan-Yuan Ma, Qiao-Qiao Yan, Rui-Nan Yuan, Si-Dian Li, Lai-Sheng Wang

**Affiliations:** 1 Department of Chemistry, Brown University, Providence, RI 02912 USA; 2 Nanocluster Laboratory, Institute of Molecular Science, Shanxi University, 030006 Taiyuan, China

**Keywords:** Chemical physics, Chemical bonding

## Abstract

Despite its electron deficiency, boron can form multiple bonds with a variety of elements. However, multiple bonds between boron and main-group metal elements are relatively rare. Here we report the observation of boron-lead multiple bonds in PbB_2_O^–^ and PbB_3_O_2_^–^, which are produced and characterized in a cluster beam. PbB_2_O^–^ is found to have an open-shell linear structure, in which the bond order of B☱Pb is 2.5, while the closed-shell [Pb≡B–B≡O]^2–^ contains a B≡Pb triple bond. PbB_3_O_2_^–^ is shown to have a Y-shaped structure with a terminal B = Pb double bond coordinated by two boronyl ligands. Comparison between [Pb≡B–B≡O]^2–^/[Pb=B(B≡O)_2_]^–^ and the isoelectronic [Pb≡B–C≡O]^–^/[Pb=B(C≡O)_2_]^+^ carbonyl counterparts further reveals transition-metal-like behaviors for the central B atoms. Additional theoretical studies show that Ge and Sn can form similar boron species as Pb, suggesting the possibilities to synthesize new compounds containing multiple boron bonds with heavy group-14 elements.

## Abstract (web summary)

Despite its electron deficiency, boron can form multiple bonds with a variety of elements, but such bonds between boron and main-group metal elements are relatively rare. Here, the authors characterize boron–lead multiple bonds in PbB_2_O^-^ and PbB_3_O_2_^-^ produced by laser vaporization.

## Introduction

Due to its electron deficiency, boron tends to form multicenter bonds in both its compounds and at nanoscales^1–5^. Boron is also capable of forming multiple chemical bonds with transition metals, such as in borylene (:BR) compounds, which usually involve a transition metal (M) with different ligands (L_*n*_), L_*n*_MBR^6^. The bonding between the metal and borylene fragment is interpreted as B→M σ-donation and M(dπ)→B back-donation^7–10^. The similarities between borylenes and carbenes (:CR_2_) suggest the bonding between boron and metal should be a double bond. However, the B–M bond lengths vary in a wide range, depending on the ligands and the R group^11^. Since the syntheses of the first transition-metal borylene complexes^12,13^, considerable progresses have been achieved in this area^14–24^. Boron-metal triple-bond characters were first suggested in [(OC)_5_CrBSiH_3_] with the B–Cr bond length of 1.871 Å^25^, slightly shorter than the B=Cr double-bond length of 1.89 Å derived from Pyykkö’s covalent radii^26^. Several transition-metal complexes with B–M bond lengths shorter than B=M double bonds have been characterized in both solid compounds and gaseous molecules^15–19^, among which some have B–M bond lengths comparable to those computed from Pyykkö’s triple-bond covalent radii. The first electron-precise transition-metal-boron triple-bond complex was identified recently by combined photoelectron spectroscopy (PES) and quantum chemistry calculations, in the linear ReB_2_O^–^ species with a B≡Re triple bond^27^. In fact, complexes with transition-metal-boron bond lengths shorter than B≡M triple bonds^28,29^ and even B≣M quadruple bonds^30,31^ have been characterized by joint gas-phase experimental and ab initio theoretical studies.

Compared with the transition-metal-boron multiple-bond complexes, compounds with multiple bonding between boron and main-group metal elements are rare^24^, even though multiple bonds of boron with light main-group elements are common. This is understandable because main-group metal elements have valence *ns* and *np* orbitals with large differences in orbital radii, decreasing the hybridization of these orbitals and making it difficult for heavy elements to form strong multiple bonds. The first molecules observed to contain main-group-metal-boron multiple bonds are the linear Bi_2_B^–^ and BiB_2_O^–^ species^32^, featuring two B=Bi double bonds in Bi_2_B^–^ and a B≡Bi triple bond in BiB_2_O^–^. Besides these, boron has only been found to form double bonds with heavy main-group elements^24,33–35^.

Here we report the observation of B–Pb multiple bonds in two molecular anions, PbB_2_O^–^ and PbB_3_O_2_^–^, by a joint PES and theoretical study. Well-resolved photoelectron spectra were obtained for these two species in the gas phase and used to elucidate their structures and bonding. Theoretical calculations and chemical bonding analyses showed that PbB_2_O^–^ has an open-shell [Pb☱B–B≡O]^–^ linear structure with a B–Pb bond order of 2.5, whereas the closed-shell [Pb≡B–B≡O]^2–^ contains a B≡Pb triple bond. The PbB_3_O_2_^–^ species has a Y-shaped structure, [Pb=B(B≡O)_2_]^–^, which consists of a B = Pb double bond coordinated by two boronyl ligands. Comparisons of the bonding in [Pb≡B–B≡O]^2–^ and [Pb=B(B≡O)_2_]^–^ with that in [Pb≡B−C≡O]^–^ and [Pb=B(C≡O)_2_]^+^ also provide evidence for transition-metal-like properties for the central B atom.

## Results and discussion

### The PES of PbB_2_O^–^

**Fig. 1:**
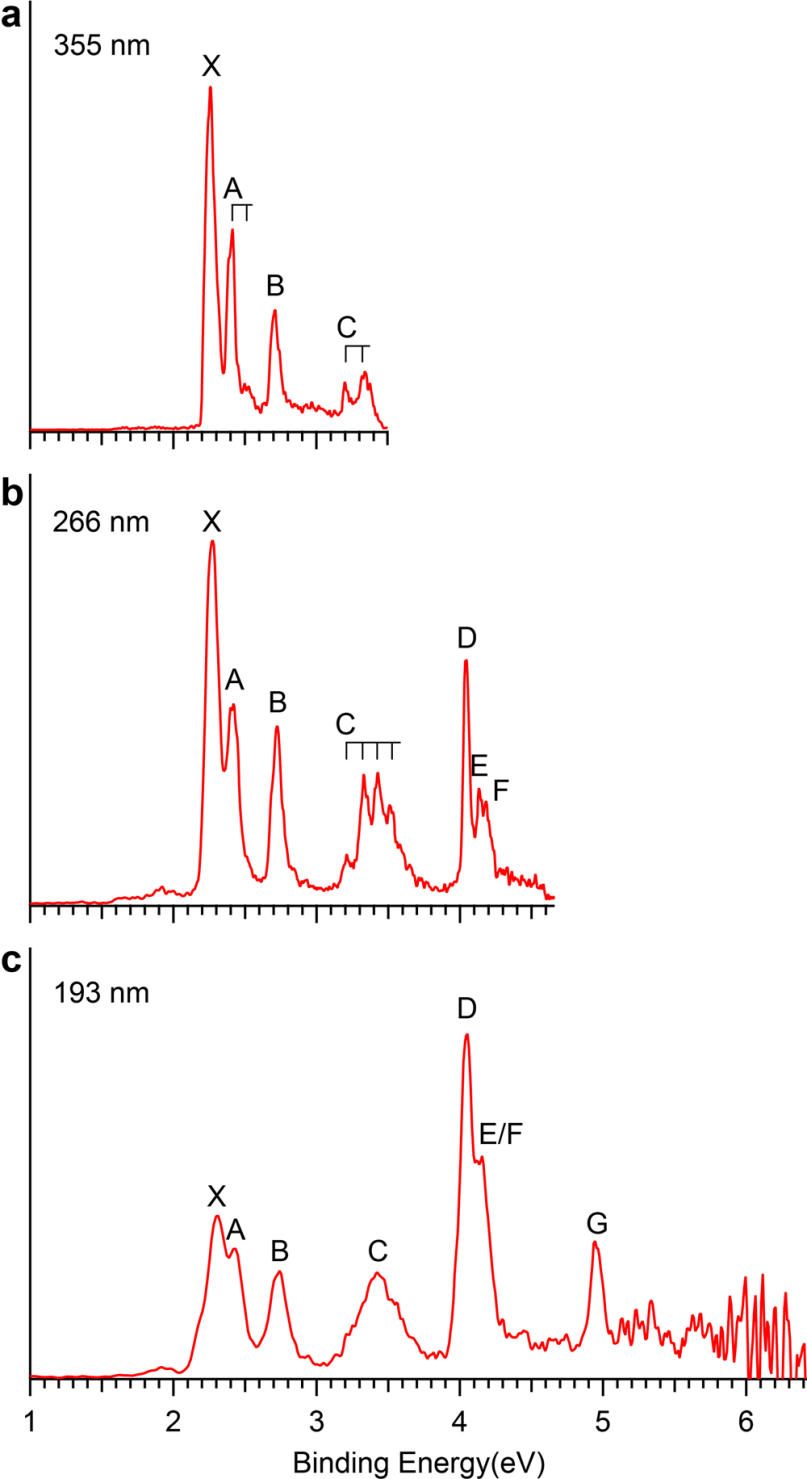
Photoelectron spectra of PbB_2_O^–^. **a** At 355 nm. **b** At 266 nm. **c** At 193 nm. The vertical lines represent resolved vibrational progressions.

The photoelectron spectra of PbB_2_O^–^ at three different photon energies are shown in Fig. 1. The spectrum at 355 nm revealed four detachment bands labeled as X, A, B, and C (Fig. 1a). The lowest binding energy band X corresponds to the detachment transition from the ground state of PbB_2_O^–^ to that of neutral PbB_2_O, whereas the higher binding energy bands represent detachment transitions to excited states of neutral PbB_2_O. Band X yielded the first vertical detachment energy (VDE) of 2.26 eV and an adiabatic detachment energy (ADE) of 2.19 eV evaluated from its onset, which also represents the electron affinity (EA) of neutral PbB_2_O. Band A was observed at 2.40 eV with a short vibrational progression with the frequency of 890 cm^−1^. Band B consisting of a single peak was observed at 2.71 eV. Two weak features labeled as C were resolved near the detachment threshold at 355 nm and they turned out to be part of a broad vibrational progression fully observed at 266 nm (Fig. 1b). The ADE and VDE for band C were measured to be 3.20 eV and 3.34 eV, respectively, and the vibrational progression yielded a frequency of 970 cm^−1^. The 266 nm spectrum also displayed a sharp peak D at 4.04 eV, closely followed by two weak peaks E (at 4.13 eV) and F (at 4.18 eV). At 193 nm (Fig. 1c), a new peak F was observed at 4.95 eV, beyond which the signal-to-noise ratios were poor and no additional PES bands could be definitively identified. The VDEs of all the observed PES bands are given in Table 1, where they are compared with theoretical results.

**Table 1:** The experimental data of PbB_2_O^–^ and their assignments.

VDE (expt.)		Configurations	Terms	VDE (MRCI)	Levels	VDE (SO)	Composition of SO coupled states
X	2.26	1σ^2^2σ^2^3σ^2^4σ^2^1π^4^5σ^2^**2π**^2^	^3^Σ^–^	2.22^a^	^3^Σ^–^_0_	2.22^a^	86.9%^3^Σ^–^ + 11.8%^1^Σ^+^ + 1.3%^3^Π
A	2.41				^3^Σ^–^_1_, ^3^Σ^–^_–1_	2.34^b^	96.9%^3^Σ^–^ + 2.2%^3^Π + 0.9%^1^Π
B	2.71		^1^Δ	2.60^c^	^1^Δ_2_	2.73^b^	92.3%^1^Δ + 7.7%^3^Π
C	3.34		^1^Σ^+^	2.90^c^	^1^Σ^+^_0_	3.17^b^	82.7%^1^Σ^+^ + 8.9%^3^Σ^–^ + 8.4%^3^Π
D	4.04	1σ^2^2σ^2^3σ^2^4σ^2^1π^4^**5σ**^**1**^2π^3^	^3^Π	3.82^c^	^3^Π_2_	4.01^b^	92.3%^3^Π + 7.7%^1^Δ
E	4.13				^3^Π_1_	4.13^b^	94.6%^3^Π + 2.8%^1^Π + 2.7%^3^Σ^–^
F	4.18				^3^Π_0_	4.33^b^	100%^3^Π
G	4.95	1σ^2^2σ^2^3σ^2^4σ^2^1π^4^**5σ**^**1**^2π^3^	^1^Π	4.61^c^	^1^Π_1_	5.07^b^	96.3%^1^Π + 3.2%^3^Π + 0.5%^3^Σ^–^

The observed features and their vertical detachment energies (VDEs) from the photoelectron spectra of PbB2O^–^ in comparison with theoretical values. All energies are given in eV.

^a^ The first VDE was calculated at the CCSD(T) level.

^b^ The higher VDEs were calculated with the SO coupling effect considered.

^c^ The higher VDEs were calculated using the MRCI approach.

### The PES of PbB_3_O_2_^–^

**Fig. 2:**
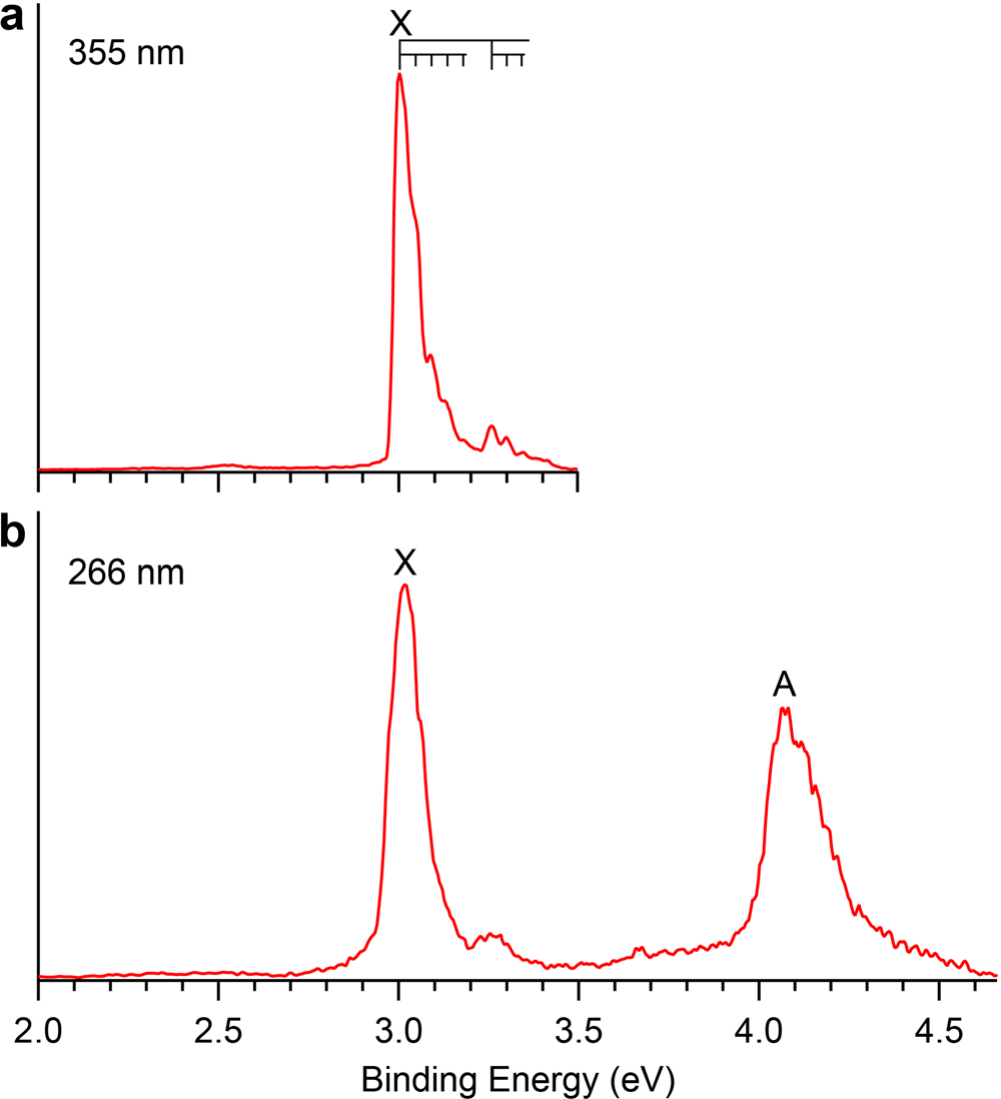
Photoelectron spectra of PbB_3_O_2_^–^. **a** At 355 nm. **b** At 266 nm. The vertical lines represent resolved vibrational progressions.

The PES spectra of PbB_3_O_2_^–^ (Fig. 2) displayed a much simpler pattern compared to that of PbB_2_O^–^. The 355 nm spectrum revealed one PES band X with partially resolved vibrational structures (Fig. 2a). Band X gave rise to an ADE of 2.96 eV and a VDE of 3.00 eV. Two vibrational progressions were discernible for band X with a high-frequency mode of 2060 cm^−1^ and a low-frequency mode of ~350 cm^−1^. Following a large energy gap, a slightly broader band A at 4.07 eV was observed at 266 nm (Fig. 2b). No higher binding energy detachment transitions were observed in the 193 nm spectrum (Supplementary Fig. 1). The observed spectroscopic data for PbB_3_O_2_^–^ are given in Table 2, where they are compared with the corresponding theoretical results.

**Table 2:** The experimental data of PbB_3_O_2_^–^ and their assignments.

Feature	VDE/*ADE*^a^ (expt.)	Configurations	Terms	VDE/*ADE*^b^ (theo.)	
				TD-PBE0	CCSD(T)
X	3.00/*2.96*	……1b_1_^2^5a_1_^2^1a_2_^2^4b_2_^2^6a_1_^2^2b_1_^1^	^2^B_1_	2.93/*2.85*^c^	3.06/*3.01*^d^
A	4.07	……1b_1_^2^5a_1_^2^1a_2_^2^4b_2_^2^6a_1_^1^2b_1_^2^	^2^A_1_	3.84^c^	3.97^d^

The observed features and their vertical detachment energies (VDEs), as well as the first adiabatic detachment energy (ADE), from the photoelectron spectra of PbB_3_O_2_^–^ in comparison with theoretical values. All energies are given in eV.

^a^ The experimentally observed VDEs and *ADE* (shown in *italic*).

^b^ The theoretically predicted VDEs and *ADE*.

^c^ The VDEs and *ADE* calculated using the TD-PBE0 method.

^d^ The first VDE and *ADE* were calculated using CCSD(T), while the second VDE was calculated using the TD-PBE0 method.

### Theoretical results

**Fig. 3:**
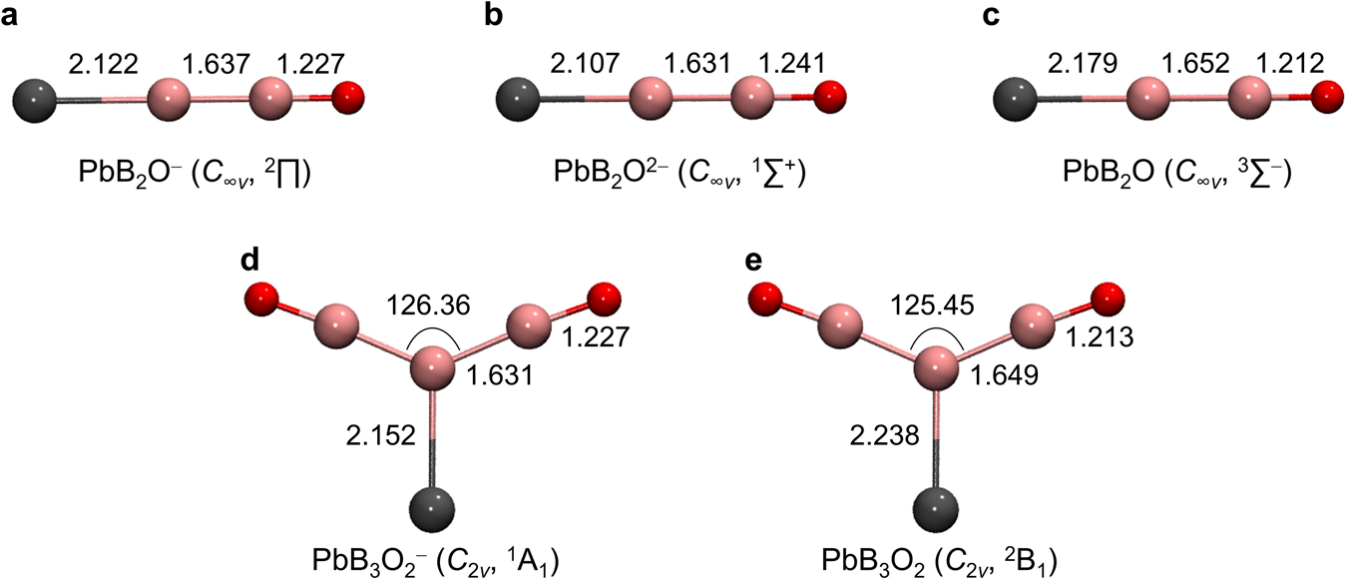
Global minimum structures of PbB_2_O and PbB_3_O_2_ at different charge states. **a** PbB_2_O^–^. **b** PbB_2_O^2–^. **c** PbB_2_O. **d** PbB_3_O_2_^–^. **e** PbB_3_O_2_. Bond lengths and bond angles are given in Å and degrees, respectively, at the CCSD/AVTZ level of theory. Cartesian coordinates of these structures are given in Supplementary Table 2.

Figure 3 depicts the global minimum (GM) structures of PbB_2_O^–^, PbB_2_O^2–^, PbB_2_O, PbB_3_O_2_^–^, and PbB_3_O_2_ at the CCSD/AVTZ level of theory^36–39^, with alternative low-lying isomers within 2.5 eV at the PBE0/AVTZ level^40^ collectively shown in Supplementary Figs. 2, 3. The GM of PbB_2_O^–^ possesses a highly stable linear structure (*C*_∞*v*_, ^2^Π), which lies 0.88 eV lower in energy than the second lowest-lying isomer PbB_2_O^–^ (*C*_*s*_, ^4^A*”*) at the PBE0/AVTZ level. It consists of a short B–Pb bond (*r*_B–Pb_ = 2.122 Å) with a BO ligand coordinated to the central B atom (Fig. 3a), similar to the previously reported linear BiB_2_O^–^ and ReB_2_O^–^ systems^27,32^. The spin-orbit (SO) coupling effect^41^ was evaluated for PbB_2_O^–^ using multi-reference configuration interaction (MRCI) calculations^42^. The SO coupling splits the ^2^Π state (electron configuration: 1*σ*^2^2*σ*^2^3*σ*^2^4*σ*^2^1*π*^4^5*σ*^2^2*π*^3^) into two sub-levels ^2^Π_3/2_ and ^2^Π_1/2_, with ^2^Π_3/2_ being lower in energy by 0.27 eV. The strong SO coupling quenches the Renner–Teller effect in the linear monoanion. Adding one electron to PbB_2_O^–^ results in the closed-shell PbB_2_O^2–^ (*C*_∞*v*_, ^1^Σ^+^), which has an even shorter B–Pb bond length (*r*_B–Pb_ = 2.107 Å) (Fig. 3b). Removing an electron from PbB_2_O^–^ leads to the triplet ground state of neutral PbB_2_O (*C*_∞*v*_, ^3^Σ^–^), as shown Fig. 3c.

PbB_3_O_2_^–^ was found to have a closed-shell Y-shaped GM (*C*_2*v*_, ^1^A_1_) featuring a terminal Pb with two BO units coordinated to the central B atom (Fig. 3d). It is 0.53 eV more stable than the second lowest-lying triplet isomer (*C*_*2v*_, ^3^A_2_) at the PBE0/AVTZ level (Supplementary Fig. 3a). The GM of neutral PbB_3_O_2_ also possesses a similar Y-shaped structure (*C*_2*v*_, ^2^B_1_) with the second isomer lying 0.61 eV higher in energy at the PBE0 level/AVTZ (Fig. 3e). The valence molecular orbitals (MOs) of PbB_2_O^–^ (*C*_∞*v*_, ^2^Π) and PbB_3_O_2_^–^ (*C*_*2v*_, ^1^A_1_) are shown in Supplementary Fig. 4.

### Comparison between the experimental and computational results

Detaching one electron from the 2*π* SOMO of PbB_2_O^–^ (Supplementary Fig. 4a) results in three final states in PbB_2_O, ^3^Σ^–^, ^1^Δ, and ^1^Σ^+^, among which the triplet ^3^Σ^–^ state with a configuration of 1*σ*^2^2*σ*^2^3*σ*^2^4*σ*^2^1*π*^4^5*σ*^2^2*π*^2^ is the ground state. The computed VDE/ADE of 2.22/2.19 eV at CCSD(T)/AVTZ are in excellent agreement with the experimental values at 2.26/2.19 eV. To interpret the higher energy VDEs, we computed the term values of the neutral excited states relative to the triplet ground state using the SA-CASSCF method with the MRCI interaction and SO coupling effects considered simultaneously. In consequence, the triplet ^3^Σ^–^ ground state splits into three closely spaced states ^3^Σ^–^_0_, ^3^Σ^–^_–1_ and ^3^Σ^–^_1_ with the calculated VDEs of 2.22, 2.34, and 2.34 eV, respectively, in excellent agreement with the observed X and A bands (Table 1). The weak vibrational peak with a spacing of 890 cm^–1^ observed for band A is due to the B–B stretching mode (ν_4_) with a computed frequency of 1019 cm^–1^ (Supplementary Fig. 5a). Franck-Condon simulations suggest that there is unresolved low-frequency Pb–B stretching vibration in band X (Supplementary Fig. 6). The next two singlet final states ^1^Δ_2_ and ^1^Σ^+^_0_ with calculated VDEs of 2.73 and 3.17 eV agree well with bands B and C at 2.71 eV and 3.34 eV, respectively. The observed frequency of 970 cm^−1^ for the vibrational progression resolved for band C is likely due to the B–B stretching mode (ν_4_), which should have a similar frequency as the ^3^Σ^–^ ground state (Supplementary Fig. 5). Detachment of one *β*-electron from the 5*σ* orbital (HOMO-1, Supplementary Fig. 4a) results in the ^3^Π neutral state, which gives rise to three SO states, ^3^Π_2_, ^3^Π_1_, and ^3^Π_0_, with calculated VDEs of 4.01, 4.13, and 4.33 eV, respectively, in agreement with the observed bands D at 4.04 eV, E at 4.13 eV, and F at 4.18 eV (Table 1). Removing one *α*-electron from the 5*σ* orbital gives rise to the ^1^Π final state with a calculated VDE of 5.07 eV, which accounts for band G at 4.95 eV. Overall, the theoretical results are in excellent agreement with the experiment, confirming the linear GM for PbB_2_O^–^.

For PbB_3_O_2_^–^, electron detachment from the 2*b*_1_ HOMO (Supplementary Fig. 4b) yields the ^2^B_1_ ground state of neutral PbB_3_O_2_. The calculated VDE/ADE of 3.06/3.01 eV at the CCSD(T)/AVTZ level are in good accord with the experimental VDE/ADE of 3.00/2.96 eV. The low-frequency vibrational progression (350 cm^–1^) corresponds to the B–Pb stretching mode with a calculated frequency of 318 cm^–1^ (*a*_1_ mode) for neutral PbB_3_O_2_ (*C*_2*v*_, ^2^B_1_), while the high-frequency mode (2060 cm^−1^) originates from the B–O stretching mode with a calculated frequency of 2026 cm^–1^ (*a*_1_ mode) (Supplementary Fig. 5b), as confirmed by the Franck-Condon simulations (Supplementary Fig. 7 and Supplementary Table 1). It should be noted that the observed frequency of 2060 cm^–1^ in PbB_3_O_2_ is similar to the previously reported B≡O symmetric stretching frequencies of 1950, 2040, 1980, and 1935 cm^–1^ in B_3_O_2_, B_4_O_2_, B_4_O_3_, and the bare BO, respectively^43–45^. The observed active vibrational modes are consistent with the geometry changes from the ground state of the anion to that of the neutral (Fig. 3) and the nature of the 2*b*_1_ HOMO that mainly involves B–Pb *π* bonding and weak B–O antibonding interactions (Supplementary Fig. 4b). The second VDE derived from electron detachment from the 6*a*_1_ HOMO-1 was calculated to be 3.84 and 3.97 eV at the PBE0 and CCSD(T) levels, respectively, consistent with band A at 4.07 eV. The 6*a*_1_ HOMO-1 is a strong B–Pb *σ* bonding orbital, consistent with the broad band A, which should contain an unresolved B–Pb stretching vibrational progression. There is a large energy gap between HOMO-2 and HOMO-1 (3.04 eV at PBE0/AVTZ level), agreeing well with the 193 nm spectrum which does not reveal any new spectral features between 4.5 and 6.4 eV (Supplementary Fig. 1). The excellent agreement between the theoretical and experimental results confirms firmly the Y-shaped GM for PbB_3_O_2_^–^.

### Multiple B–Pb bonding in PbB_2_O^–/2–^ and PbB_3_O_2_^–^

**Fig. 4:**
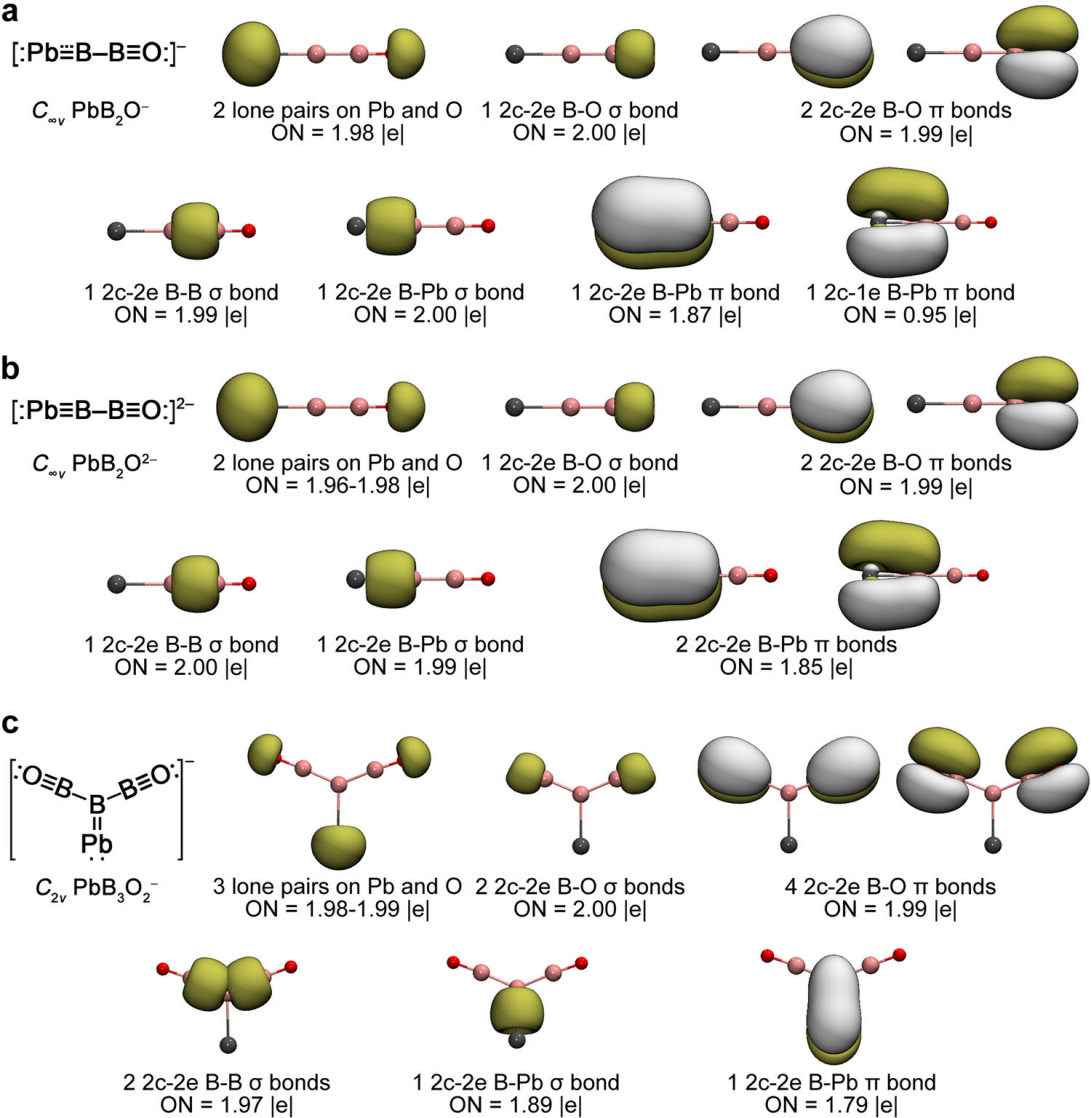
Chemical bonding anaylses using AdNDP. **a** PbB_2_O^–^. **b** PbB_2_O^2–^. **c** PbB_3_O_2_^–^. The occupation numbers (ONs) are indicated. The corresponding Lewis electronic structures are also depicted.

Adaptive natural density partitioning (AdNDP)^46^ analyses were performed on PbB_2_O^–^, PbB_2_O^2–^, and PbB_3_O_2_^–^ to understand the nature of their chemical bonding. AdNDP basically transforms the MOs into more familiar bonding elements, such as lone pairs, two-center two-electron (2c-2e) bonds or multicenter delocalized bonds. As shown in Fig. 4a, the linear PbB_2_O^–^ possesses one Pb 6 *s* and one O lone pairs and a B≡O triple bond in the first row. The second row depicts one 2c-2e B–B *σ* bond, one 2c-2e B–Pb *σ* bond, one 2c-2e B–Pb *π* bond, and one 2c-1e B–Pb *π* bond, giving rise to a B–Pb bond order of 2.5. Thus, there is no *sp* hybridization in Pb, which uses its three valence 6*p* orbitals to form a triple bond with the central B atom. Both B atoms undergo *sp* hybridization and form triple bonds. The B–Pb bond length of 2.122 Å lies between the B=Pb double-bond length (2.13 Å) and the B≡Pb triple-bond length (2.10 Å) predicted from Pyykkö’s covalent atomic radii^25^, consistent with the 2.5 bond order for the B–Pb multiple bond. Hence, the linear PbB_2_O^–^ species can be described as [Pb☱B–B≡O]^–^. Adding one electron to PbB_2_O^–^ yields the closed-shell and electron-precise PbB_2_O^2–^ which contains an ideal Pb≡B triple bond including one 2c-2e Pb–B *σ* bond and two degenerate 2c-2e Pb–B *π* bonds, *i.e*. [Pb≡B–B≡O]^2–^, as detailed in the second row of Fig. 4b. The B–Pb bond length in PbB_2_O^2–^ is reduced to 2.107 Å, in good accord with the B≡Pb triple-bond length (2.10 Å) derived from Pyykkö’s triple-bond covalent atomic radii^26^.

The central B atom in PbB_3_O_2_^–^ (Fig. 3d) undergoes *sp*^2^ hybridization and the AdNDP bonding analyses of the electron-precise monoanion are straightforward (Fig. 4c). It contains one Pb 6 *s* lone pair, two O lone pairs, and two B≡O boronyl ligands in the first row. The second row displays two 2c-2e B–B *σ* bonds between the central B and two BO ligands, one 2c-2e B–Pb *σ* bond, and one 2c-2e B–Pb *π* bond, giving rise to a terminal Pb=B double bond. The B–Pb bond length of 2.152 Å is comparable to the B=Pb double-bond length of 2.13 Å from Pyykkö’s covalent radii^26^. PbB_3_O_2_^–^ can thus be formulated as [Pb=B(B≡O)_2_]^–^. Natural resonance theory (NRT) analyses^47,48^ at the PBE0/AVTZ level give rise to a B–Pb bond order of 2.39 for PbB_2_O^–^, 2.84 for PbB_2_O^2–^, and 1.84 for PbB_3_O_2_^–^, consistent with the bond order designations of 2.5, 3, and 2 for these species on the basis of electron counts, respectively. The covalency of the Pb–B multiple bonds can be understood from the similar electronegativities of Pb (2.33) and B (2.04). The PbB_2_O^–^, PbB_2_O^2–^ and PbB_3_O_2_^–^ species are the first experimentally confirmed molecules with B–Pb multiple bonds. We have also calculated MB_2_O^–/2–^ and MB_3_O_2_^–^ for M=Ge and Sn and found that both the Ge and Sn species have similar GM structures and bonding patterns as their Pb counterparts (see Supplementary Figs. 8–10).

### Transition-metal-like behaviors of the central boron in [Pb≡B−CO]^−^ and [Pb=B(CO)_2_]^+^

The electron deficiency of boron has led to novel structures and bonding in various boron compounds. Recently, boron has been shown to exhibit “metallomimetic” properties^49^, such as the formation of stable borylene dicarbonyl complex, in which two CO ligands are coordinated to a monovalent boron via donor-acceptor bonds^50^, a prototypical transition-metal behavior. Another unexpected metallomimetic property of boron is its capability to form a half-sandwich complex with the aromatic B_7_^3−^ ligand in the recently observed [(*η*^7^-B_7_)-B-BO]^−^ species^51^. Since BO^−^ is isoelectronic with CO, we optimized the geometric and electronic structures of the linear [Pb≡B−C≡O]^–^ and Y-shaped [Pb=B(C≡O)_2_]^+^ and found that these carbonyl complexes exhibit similar AdNDP bonding patterns as their boronyl counterparts [Pb≡B–B≡O]^2–^ and [Pb=B(B≡O)_2_]^–^, respectively (see Fig. 4 and Supplementary Fig. 11), with the two 2c-2e B–Pb *π* bonds in [Pb≡B–B≡O]^2–^ (Fig. 4b) changed to two 3c-2e Pb-B-C *π* bonds in [Pb≡B−C≡O]^–^ (Supplementary Fig. 11a) and the 2c-2e B−Pb *π* bond in [Pb=B(B≡O)_2_]^–^ (Fig. 4c) extended to a 4c-2e Pb-B-C2 *π* bond in [Pb=B(C≡O)_2_]^+^ (Supplementary Fig. 11b). These carbonyl complexes may be more viable for chemical syntheses if a suitable ligand can be found to coordinate to Pb.

In conclusion, we have characterized the first B–Pb multiple bonds in the PbB_2_O^–/2–^ and PbB_3_O_2_^–^ molecular anions, using photoelectron spectroscopy and ab initio calculations. Excellent agreement between the theoretical and experimental data confirms that the global minimum of PbB_2_O^–^ has an open-shell linear structure with a B–Pb bond order of 2.5, whereas the closed-shell PbB_2_O^2–^ contains a B≡Pb triple bond. The PbB_3_O_2_^–^ species has been shown to have a Y-shaped structure with a terminal B=Pb double bond and two BO ligands coordinated to the central B atom. Theoretical calculations indicate that MB_2_O^–/2–^ and MB_3_O_2_^–^ with M=Ge and Sn display similar structures and bonding as the Pb counterparts. It is further revealed that [Pb≡B–B≡O]^2–^ and [Pb=B(B≡O)_2_]^–^ have similar bonding patterns with their isoelectronic carbonyl complexes [Pb≡B–C≡O]^–^ and [Pb=B(C≡O)_2_]^+^, opening the door to design main-group-metal-boron complexes with multiple bonds, as well as new boron metallomemetic compounds.

## Methods

### Photoelectron spectroscopy

The PES experiments were carried out using a magnetic-bottle apparatus with a laser vaporization supersonic cluster source^4,52^. The PbB_2_O^–^ and PbB_3_O_2_^–^ clusters were produced by laser vaporization of a Pb/^10^B mixed target inside a clustering nozzle. The laser-induced plasma was cooled by a He carrier gas seeded with 5% Ar, initiating nucleation in the nozzle. The O-containing clusters were formed due to the residue oxygen impurity on the target surface. Clusters formed inside the nozzle were entrained in the carrier gas and underwent a supersonic expansion. Negatively charged clusters were extracted perpendicularly from the collimated cluster beam and analyzed by a time-of-flight mass spectrometer. The PbB_2_O^–^ and PbB_3_O_2_^–^ clusters were mass-selected and decelerated before photodetachment. Three photon energies were used in the current experiment, including 355 nm (3.496 eV) and 266 nm (4.661 eV) from a Nd:YAG laser, and 193 nm from an ArF excimer laser. The photoelectron spectra were calibrated using the known spectra of Bi^–^. The electron kinetic energy (*E*_*k*_) resolution of the magnetic-bottle electron analyzer (Δ*E*_*k*_/*E*_*k*_) was around 2.5%, that is, about 25 meV for photoelectrons with 1 eV kinetic energy.

### Computational methods

The global minima of PbB_2_O^–^, PbB_2_O^2–^, PbB_2_O, PbB_3_O_2_^–^, and PbB_3_O_2_ were examined using the unbiased Coalescence Kick (CK) global search method^53^ at the PBE0/lanl2dz^40,54–56^ level of theory with different spin multiplicities. Low-lying isomers were further refined using the PBE0 functional with the aug-cc-pVTZ-pp basis set and the ECP60MDF relativistic effective core potential for Pb and the aug-cc-pVTZ basis sets for B and O (abbreviated as AVTZ)^36,37^. Vibrational analyses were performed at the same level to ensure that all the optimized structures were true minima. The lowest-lying isomer for each species was re-optimized at CCSD/AVTZ level^38,39^ to obtain more reliable structures and more accurate energies. The first ADEs and VDEs for the global minima of PbB_2_O^–^ and PbB_3_O_2_^–^ were calculated at the PBE0/AVTZ and CCSD(T)^57–59^/AVTZ//PBE0/AVTZ [abbreviated as CCSD(T)/AVTZ] levels. The ADE in each case was calculated as the energy difference between the anion and neutral species at their respective optimized geometries. For PbB_2_O^–^, the first VDE was calculated as the energy differences between the doublet ground state (^2^Π) of the anion and the lowest-lying triplet state (^3^Σ^–^) of the neutral species at the optimized anion geometry. The energies for higher excited states of neutral PbB_2_O were computed using the state-averaged (SA) complete active space self-consistent field (CASSCF) method^60,61^ with the AVTZ basis set. An active space with 8 electrons and 9 orbitals was chosen for PbB_2_O. Both multi-reference configuration interaction^42^ (MRCI) and spin-orbit (SO) coupling^41^ were considered in these calculations. The ^3^Σ^–^, ^1^Δ, ^1^Σ^+^, ^3^Π, and ^1^Π states of neutral PbB_2_O were included in the SA8-CASSCF(8,9) calculations. For PbB_3_O_2_^–^, the first VDE was calculated as the energy difference between the singlet ground state of the anion and the lowest doublet state of the neutral molecule at the anion geometry. Higher VDEs were calculated using the TD-DFT method^62^ at the PBE0/AVTZ level. Vibrational frequencies of the ground states of neutral PbB_2_O and PbB_3_O_2_ were calculated at the CCSD/AVTZ level. Chemical bonding analyses were done using the adaptive natural density partitioning (AdNDP) approach^46^ at the PBE0/AVTZ level of theory. Franck−Condon simulations were performed using ezSpectrum^63^ at the CCSD/AVTZ level of theory. All the PBE0, CCSD, and CCSD(T) calculations were performed using the Gaussian 09 program^64^, while the CASSCF and MRCI calculations were done employing the Molpro 2012 code^65^.

## Supplementary information

Supplemental Information

## Data Availability

The data that support the findings of this study are available within the article and the associated Supplementary Information. Any other data are available from the corresponding authors upon request.

## Code availability

All codes used in this study are commercially available or available from the authors cited in the references.
