# Supplementary material for: Boron-lead multiple bonds in the PbB2O– and PbB3O2– clusters
Source: Commun Chem. 2022 Mar 3;5:25. doi: 10.1038/s42004-022-00643-1 (PMC9814552; doi:10.1038/s42004-022-00643-1)
Supplement: Supplementary file 1 — Supplemental Information [file 42004_2022_643_MOESM1_ESM.pdf]

Supplementary Information

Boron-Lead Multiple Bonds in the PbB_2O^- and $PbB_3O_2^-$ Clusters

Wei-Jia Chen[1,†], Teng-Teng Chen[1,†], Qiang Chen[2,†*], Hai-Gang Lu[2], Xiao-Yun Zhao[2], Yuan-Yuan Ma[2], Qiao-Qiao Yan[2], Rui-Nan Yuan[2], Si-Dian Li[2*] & Lai-Sheng Wang[1*]

[1]Department of Chemistry, Brown University, Providence, Rhode Island 02912, United States

[2]Nanocluster Laboratory, Institute of Molecular Science, Shanxi University, Taiyuan 030006, China

[†]These authors contributed equally: Wei-Jia Chen, Teng-Teng Chen, Qiang Chen

*Email: chenqiang@sxu.edu.cn; lisidian@sxu.edu.cn; lai-sheng_wang@brown.edu

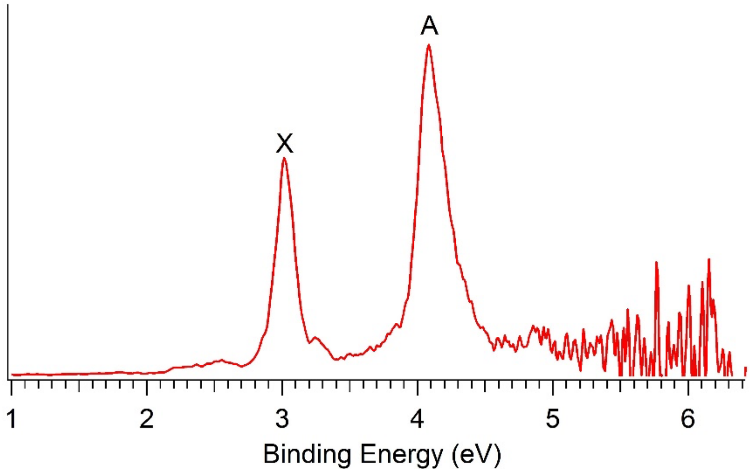

Supplementary Fig. 1. Photoelectron spectrum of $PbB_3O_2^-$ at 193 nm.

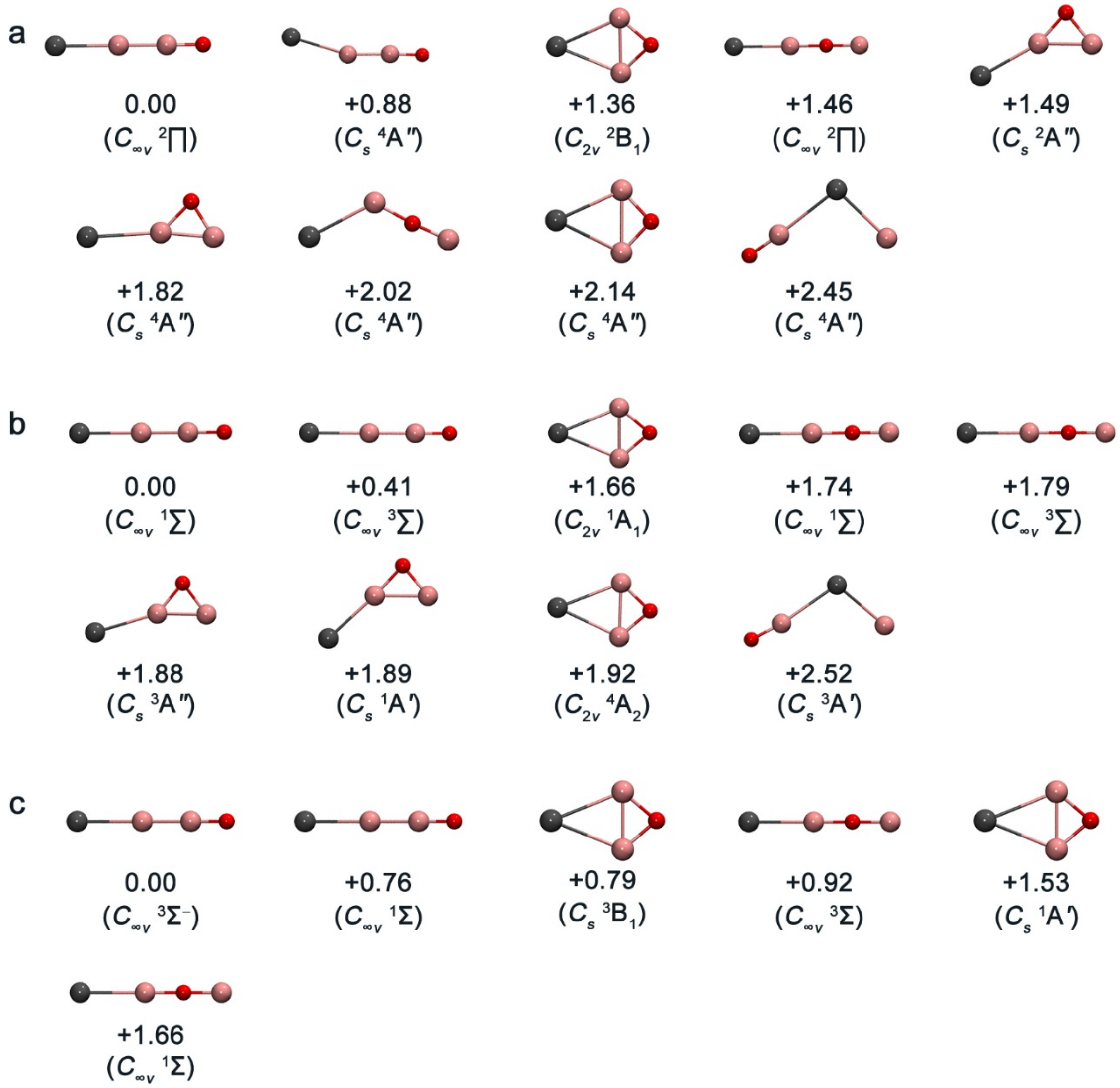

Supplementary Fig. 2. Low-lying isomers of (a) PbB_2O^-, (b) PbB_2O^{2-}, and (c) PbB_2O at the PBE0/AVTZ level of theory, along with their point group symmetries and electronic sates. Relative energies are given in eV.

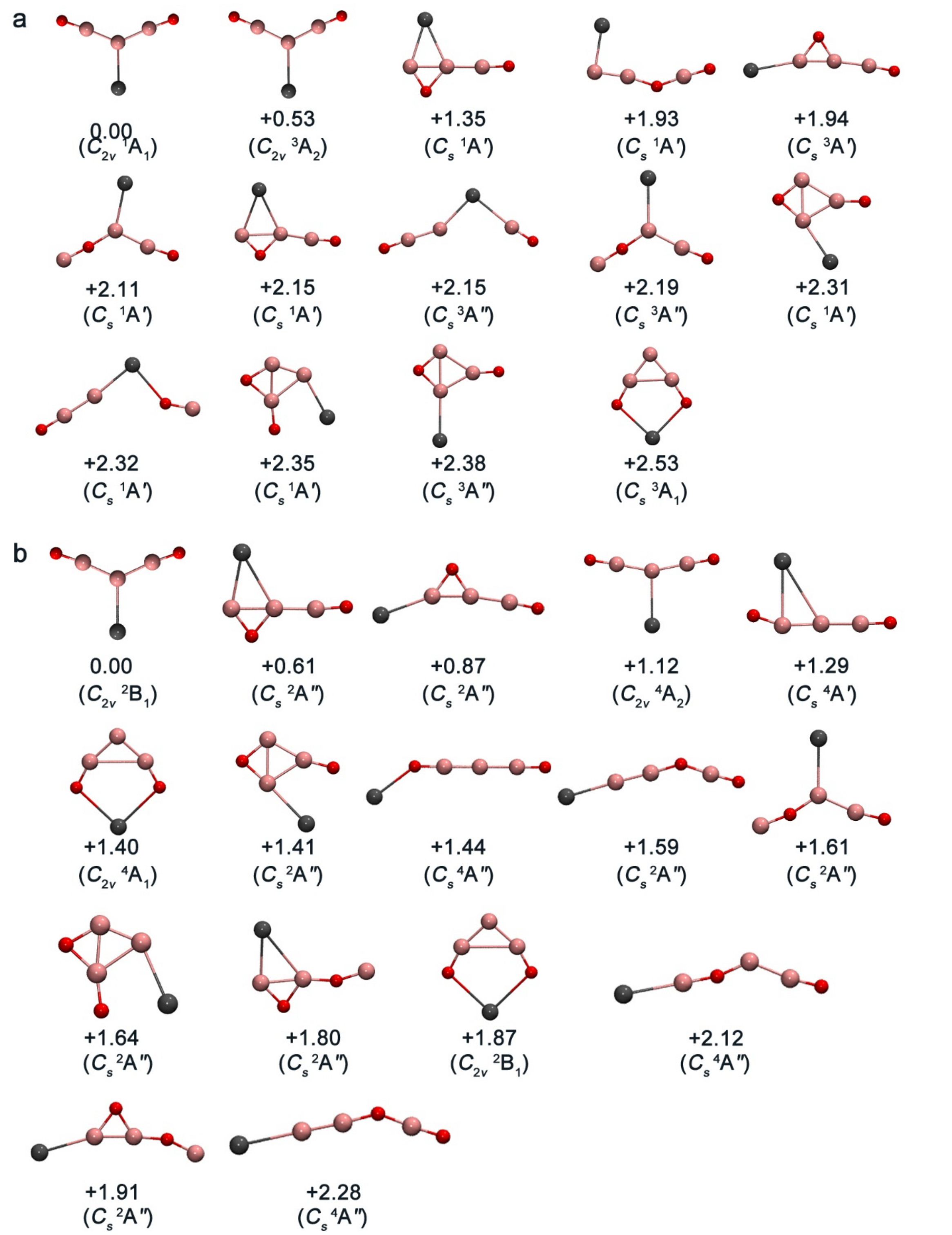

Supplementary Fig. 3. Low-lying isomers of (a) $PbB_3O_2^-$ and (b) PbB_3O_2 at the PBE0/AVTZ level of theory, along with their point group symmetries and electronic sates. Relative energies are given in eV.

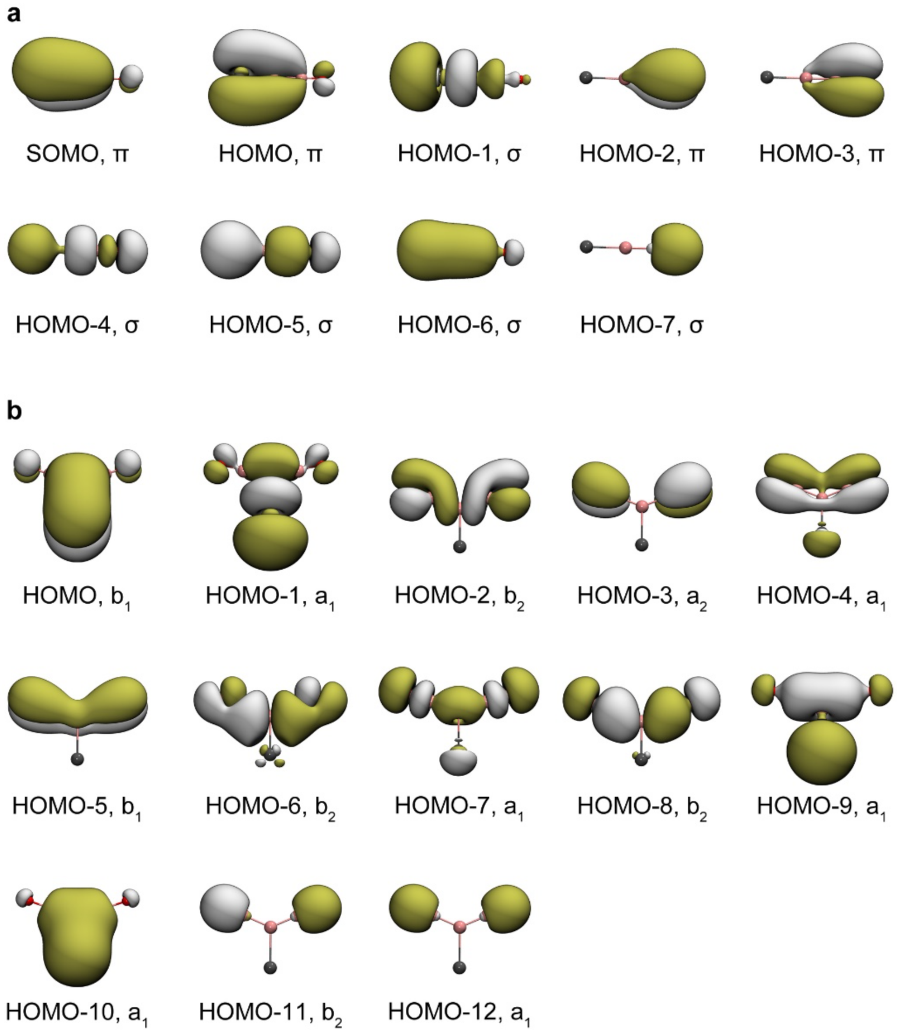

Supplementary Fig. 4. Occupied valence molecular orbitals for the global minima of (a) PbB_2O^- ($C_{\infty v}$, $^2\Pi$) and (b) $PbB_3O_2^-$ (C_{2v}, $^1A_1$).

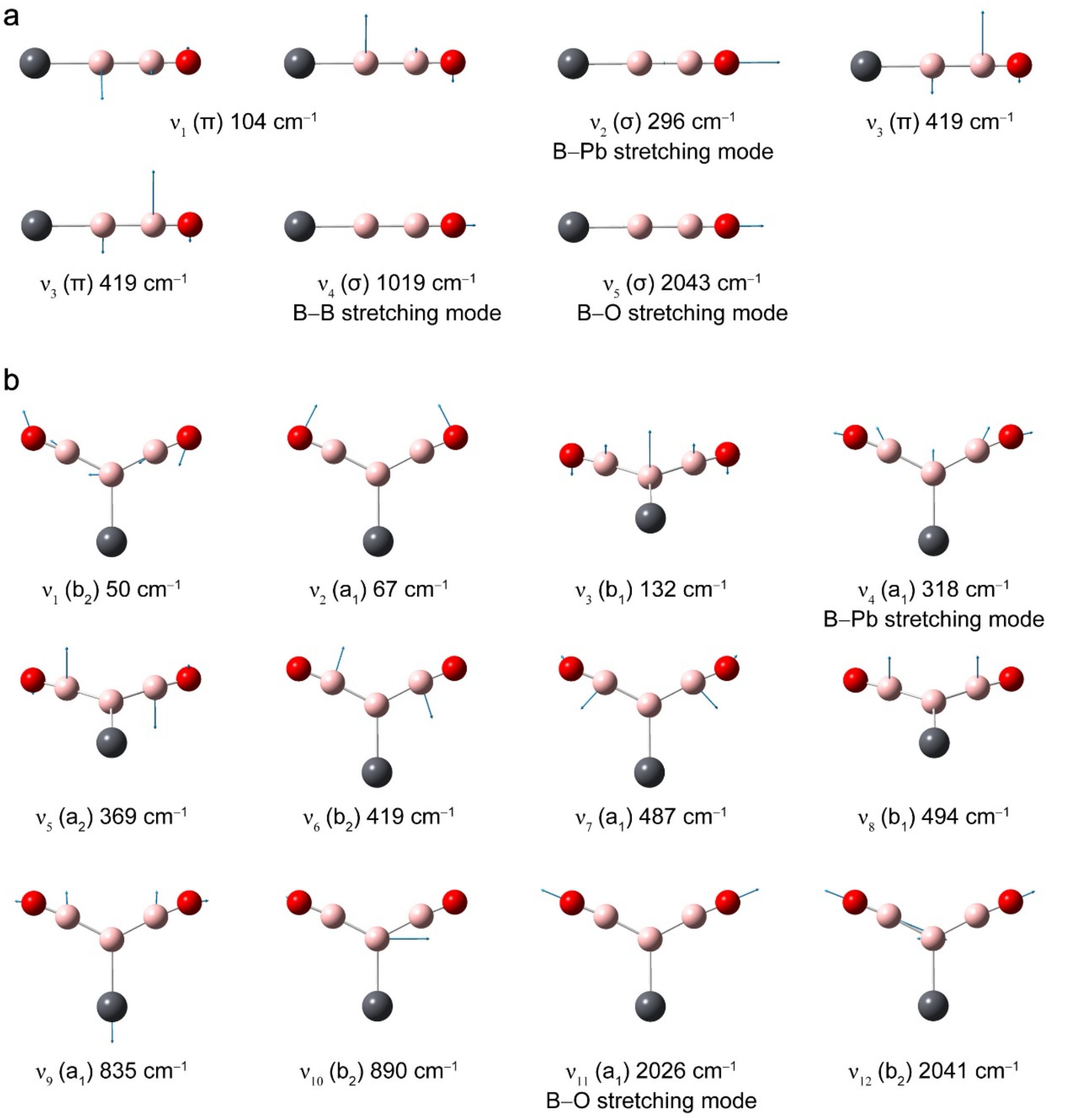

Supplementary Fig. 5. The normal modes and their symmetries for the ground electronic states of (a) PbB_2O ($C_{\infty v}$, $^3\Sigma^-$) and (b) PbB_3O_2 (C_{2v}, $^2B_1$) at the CCSD/AVTZ level. Isotopes ^{10}B and ^{207}Pb were used in the calculations.

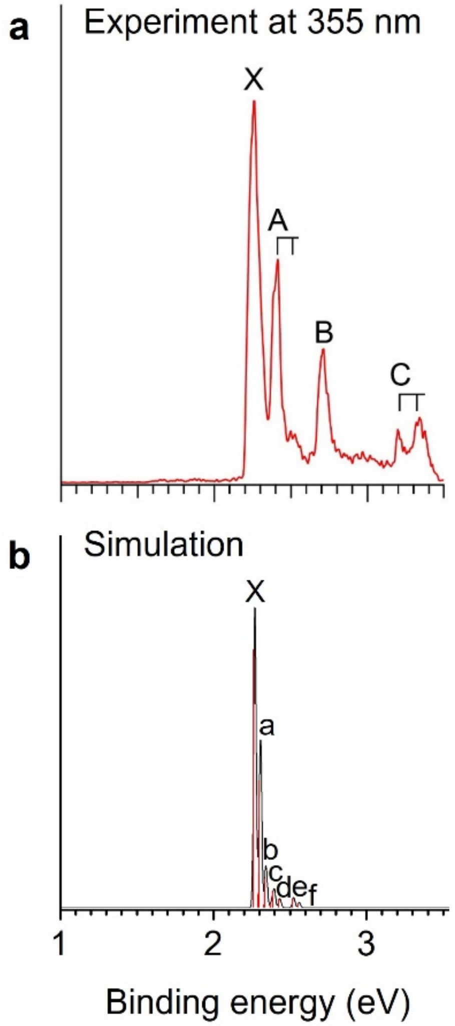

Supplementary Fig. 6. Comparison between (a) the photoelectron spectrum of PbB_2O^- at 355 nm and (b) the simulated spectrum at the CCSD/AVTZ level of theory. The Franck−Condon simulation was performed using ezSpectrum. Isotopes ^{10}B and ^{207}Pb were used in the calculation. Peaks *a* and *b* in (b) represent the Pb−B vibrational progression; peaks *d* and *e* indicate the B−B vibrational progression.

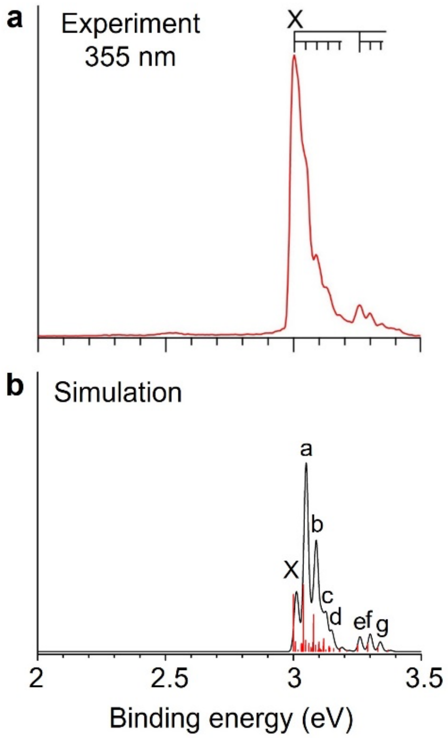

Supplementary Fig. 7. Comparison between (a) the photoelectron spectrum of $PbB_3O_2^-$ at 355 nm and (b) the simulated spectrum at the CCSD/AVTZ level of theory. The Franck−Condon simulation was performed using ezSpectrum. Isotopes ^{10}B and ^{207}Pb were used in the calculation.

Supplementary Table 1. Observed vibrational peaks in the 355 nm photoelectron spectrum of $PbB_3O_2^-$ and comparison with the calculated frequencies computed at the CCSD/AVTZ level of theory. Isotopes ^{10}B and ^{207}Pb were used in calculation.

peak	Observed vibrational peak (eV)	Calculated vibrational peak (eV)	Calculated vibrational frequency (cm^{-1})
X	3.00		
a	3.04	3.04	318
b	3.09	3.08	637
c	3.13	3.10	806
d	3.17	3.14	1153
e	3.26	3.25	2026
f	3.30	3.29	2345
g	3.34	3.33	2663

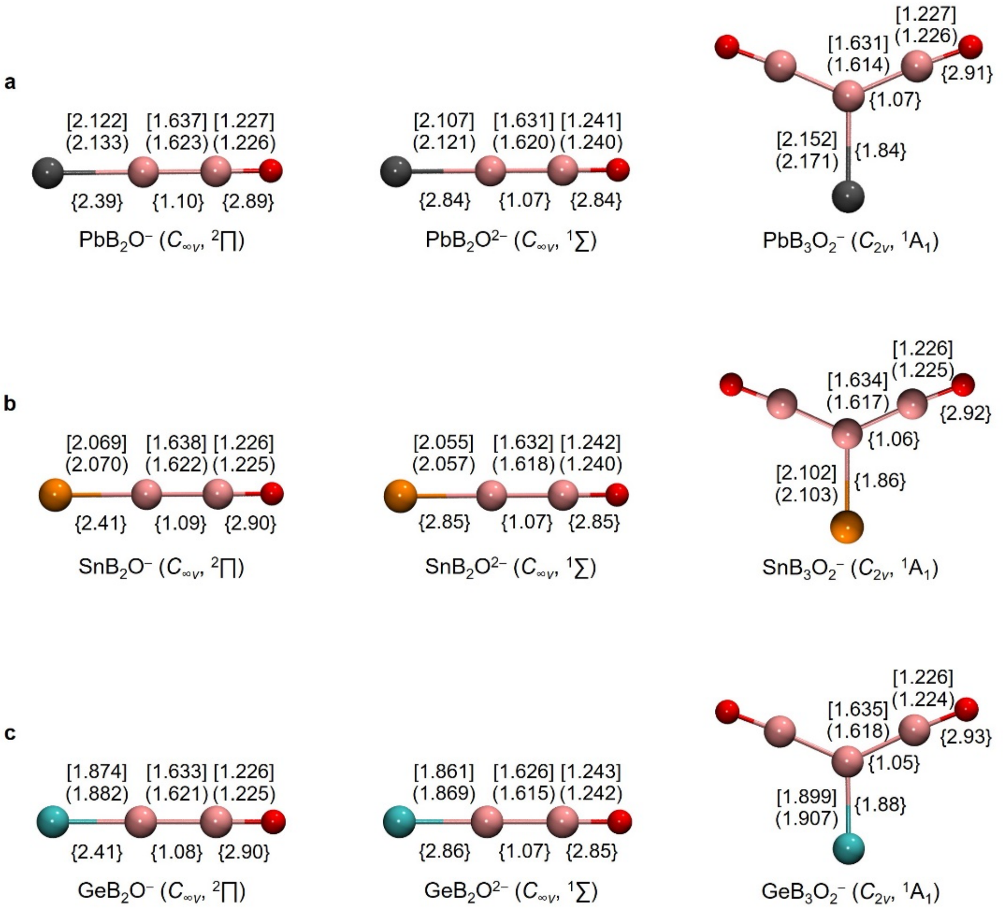

Supplementary Fig. 8. The global minimum structures of MB_2O^-, MB_2O^{2-} and $MB_3O_2^-$ for M = Pb (a), Sn (b), Ge (c) at the PBE0 and CCSD levels. The aug-cc-pVTZ-pp basis set and the ECP60MDF (Pb), ECP28MDF (Sn), and ECP10MDF (Ge) relativistic effective core potentials (ECP) were used for Pb, Sn, and Ge. The aug-cc-pVTZ basis set was used for B and O. The values in parentheses and square brackets represent the bond lengths (Å) at the PBE0 and CCSD levels, respectively. The values in braces show the natural resonance theory (NRT) bond orders at the PBE0 level.

At the CCSD level, the calculated B–M (M = Pb/Sn/Ge) bond lengths in MB_2O^{2-} (Pb/Sn/Ge: 2.107/2.055/1.861 Å) and $MB_3O_2^-$ (Pb/Sn/Ge: 2.152/2.102/1.899 Å) are consistent with the B≡M triple-bond lengths (Pb/Sn/Ge: 2.10/2.05/1.87 Å) and B=M double-bond lengths (Pb/Sn/Ge: 2.13/2.08/1.89 Å), respectively, derived from Pyykkö's covalent atomic radii. The calculated B–M (M = Pb/Sn/Ge) bond lengths in MB_2O^- (Pb/Sn/Ge: 2.122/2.069/1.874 Å) fall in between the B≡M triple-bond and the B=M double-bond lengths. At the PBE0 level, the computed B–M bond orders (M = Pb/Sn/Ge) of MB_2O^- (Pb/Sn/Ge: 2.39/2.41/2.41), MB_2O^{2-} (Pb/Sn/Ge: 2.84/2.85/2.86), and $MB_3O_2^-$ (Pb/Sn/Ge: 1.84/1.86/1.88) are close to 2.5, 3.0, and 2.0, respectively. These data are consistent with the calculated B–M bond lengths at the CCSD level.

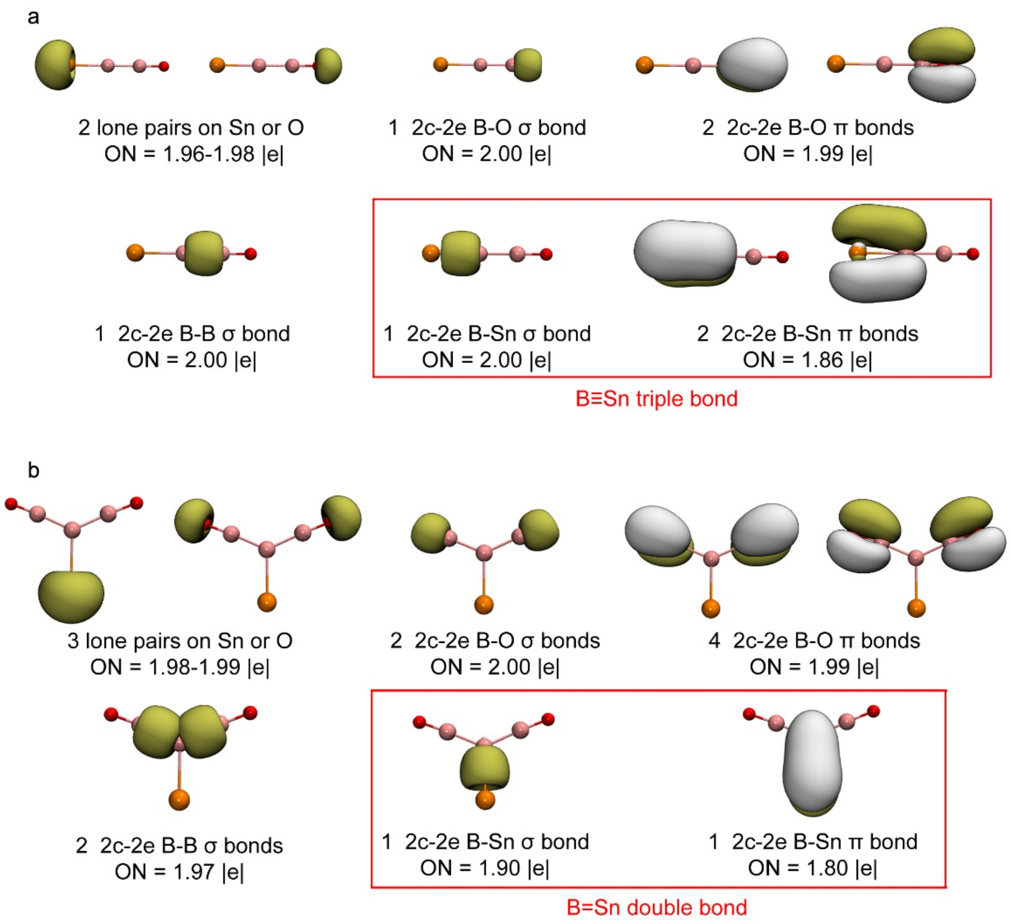

Supplementary Fig. 9. AdNDP analyses of (a) SnB_2O^{2-} and (b) $SnB_3O_2^{-}$ at the PBE0/AVTZ level. Occupation numbers (ON) are shown. The B≡Sn triple bond in SnB_2O^{2-} and the B=Sn double bond in $SnB_3O_2^{-}$ are highlighted.

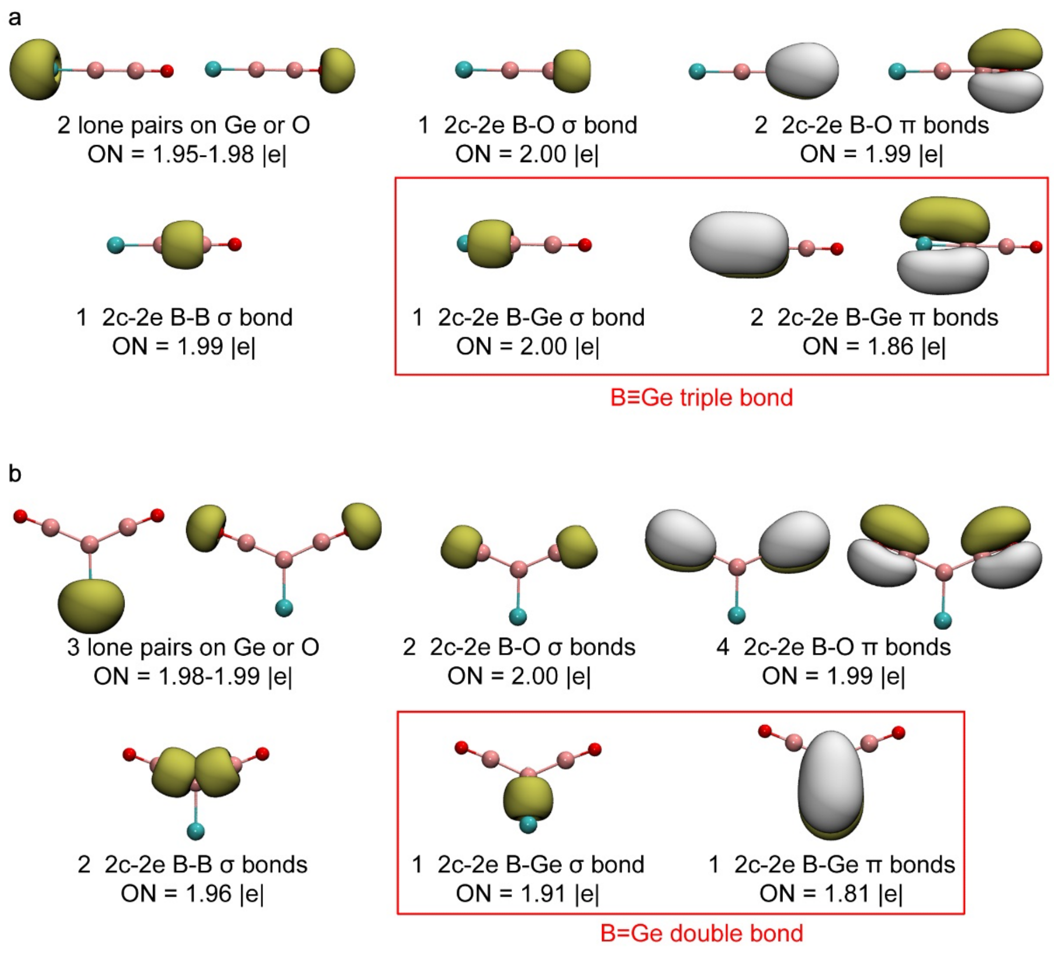

Supplementary Fig. 10. AdNDP analyses of (a) GeB_2O^{2-} and (b) $GeB_3O_2^-$ at the PBE0/AVTZ level. Occupation numbers (ON) are shown. The B≡Ge triple bond in GeB_2O^{2-} and the B=Ge double bond in $GeB_3O_2^-$ are highlighted in boxes.

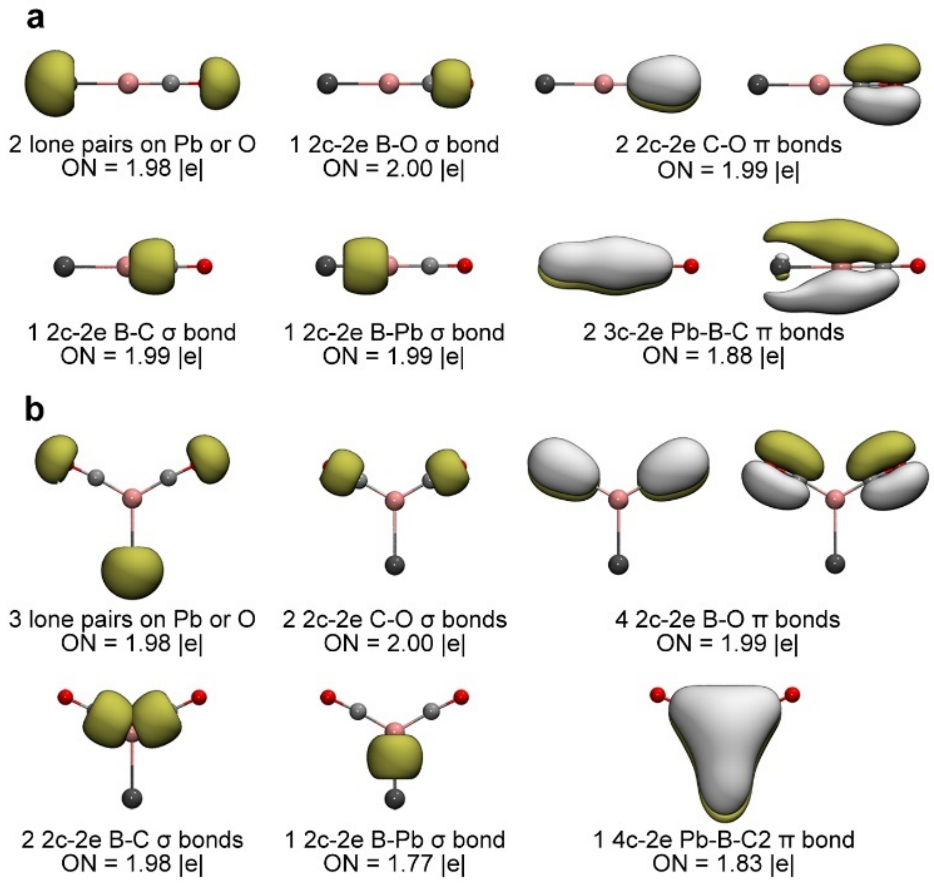

Supplementary Fig. 11. AdNDP bonding patterns of (a) $[Pb{\equiv}B{-}C{\equiv}O]^{-}$ and (b) $[Pb{=}B(C{\equiv}O)_2]^{+}$.

Supplementary Table 2. Cartesian coordinates of CCSD/AVTZ-optimized structures shown in Figure 3.

a PbB_2O^- ($C_{\infty v}$, $^2\Pi$)

Pb	0.00000000	0.00000000	0.69287300
B	0.00000000	0.00000000	-1.42875800
B	0.00000000	0.00000000	-3.06603600
O	0.00000000	0.00000000	-4.29270400

b PbB_2O^{2-} ($C_{\infty v}$, $^1\Sigma^+$)

Pb	0.00000000	0.00000000	0.69066700
B	0.00000000	0.00000000	-1.41626700
B	0.00000000	0.00000000	-3.04772100
O	0.00000000	0.00000000	-4.28934900

c PbB_2O ($C_{\infty v}$, $^3\Sigma^-$)

Pb	0.00000000	0.00000000	0.70378500
B	0.00000000	0.00000000	-1.47473500
B	0.00000000	0.00000000	-3.12637000
O	0.00000000	0.00000000	-4.33811000

d $PbB_3O_2^-$ (C_{2v}, $^1A_1$)

Pb	0.00000000	0.00000000	0.83004700
B	0.00000000	0.00000000	-1.32219300
B	0.00000000	1.45522100	-2.05792300
B	0.00000000	-1.45522100	-2.05792300
O	0.00000000	2.57726700	-2.55460300
O	0.00000000	-2.57726700	-2.55460300

e PbB_3O_2 (C_{2v}, $^2B_1$)

Pb	0.00000000	0.00000000	0.85296400
B	0.00000000	0.00000000	-1.38547700
B	0.00000000	1.46522400	-2.14090700
B	0.00000000	-1.46522400	-2.14090700
O	0.00000000	2.58787900	-2.60041000
O	0.00000000	-2.58787900	-2.60041000
